# Distinguishing case study as a research method from case reports as a publication type

**DOI:** 10.5195/jmla.2019.615

**Published:** 2019-01-01

**Authors:** Kristine M. Alpi, John Jamal Evans

**Affiliations:** OHSU Library, Oregon Health & Science University, Portland, OR, krisalpi@gmail.com; North Carolina Community College System, Raleigh, NC, jevans@nccommunitycolleges.edu

## Abstract

The purpose of this editorial is to distinguish between case reports and case studies. In health, case reports are familiar ways of sharing events or efforts of intervening with single patients with previously unreported features. As a qualitative methodology, case study research encompasses a great deal more complexity than a typical case report and often incorporates multiple streams of data combined in creative ways. The depth and richness of case study description helps readers understand the case and whether findings might be applicable beyond that setting.

Single-institution descriptive reports of library activities are often labeled by their authors as “case studies.” By contrast, in health care, single patient retrospective descriptions are published as “case reports.” Both case reports and case studies are valuable to readers and provide a publication opportunity for authors. A previous editorial by Akers and Amos about improving case studies addresses issues that are more common to case reports; for example, not having a review of the literature or being anecdotal, not generalizable, and prone to various types of bias such as positive outcome bias [1]. However, case study research as a qualitative methodology is pursued for different purposes than generalizability. The authors’ purpose in this editorial is to clearly distinguish between case reports and case studies. We believe that this will assist authors in describing and designating the methodological approach of their publications and help readers appreciate the rigor of well-executed case study research.

Case reports often provide a first exploration of a phenomenon or an opportunity for a first publication by a trainee in the health professions. In health care, case reports are familiar ways of sharing events or efforts of intervening with single patients with previously unreported features. Another type of study categorized as a case report is an “N of 1” study or single-subject clinical trial, which considers an individual patient as the sole unit of observation in a study investigating the efficacy or side effect profiles of different interventions. Entire journals have evolved to publish case reports, which often rely on template structures with limited contextualization or discussion of previous cases. Examples that are indexed in MEDLINE include the *American Journal of Case Reports*, *BMJ Case Reports, Journal of Medical Case Reports,* and *Journal of Radiology Case Reports*. Similar publications appear in veterinary medicine and are indexed in CAB Abstracts, such as *Case Reports in Veterinary Medicine* and *Veterinary Record Case Reports*.

As a qualitative methodology, however, case study research encompasses a great deal more complexity than a typical case report and often incorporates multiple streams of data combined in creative ways. Distinctions include the investigator’s definitions and delimitations of the case being studied, the clarity of the role of the investigator, the rigor of gathering and combining evidence about the case, and the contextualization of the findings. Delimitation is a term from qualitative research about setting boundaries to scope the research in a useful way rather than describing the narrow scope as a limitation, as often appears in a discussion section. The depth and richness of description helps readers understand the situation and whether findings from the case are applicable to their settings.

## CASE STUDY AS A RESEARCH METHODOLOGY

Case study as a qualitative methodology is an exploration of a time- and space-bound phenomenon. As qualitative research, case studies require much more from their authors who are acting as instruments within the inquiry process. In the case study methodology, a variety of methodological approaches may be employed to explain the complexity of the problem being studied [2, 3].

Leading authors diverge in their definitions of case study, but a qualitative research text introduces case study as follows:

Case study research is defined as a qualitative approach in which the investigator explores a real-life, contemporary bounded system (a case) or multiple bound systems (cases) over time, through detailed, in-depth data collection involving multiple sources of information, and reports a case description and case themes. The unit of analysis in the case study might be multiple cases (a multisite study) or a single case (a within-site case study). [4]

Methodologists writing core texts on case study research include Yin [5], Stake [6], and Merriam [7]. The approaches of these three methodologists have been compared by Yazan, who focused on six areas of methodology: epistemology (beliefs about ways of knowing), definition of cases, design of case studies, and gathering, analysis, and validation of data [8]. For Yin, case study is a method of empirical inquiry appropriate to determining the “how and why” of phenomena and contributes to understanding phenomena in a holistic and real-life context [5]. Stake defines a case study as a “well-bounded, specific, complex, and functioning thing” [6], while Merriam views “the case as a thing, a single entity, a unit around which there are boundaries” [7].

Case studies are ways to explain, describe, or explore phenomena. Comments from a quantitative perspective about case studies lacking rigor and generalizability fail to consider the purpose of the case study and how what is learned from a case study is put into practice. Rigor in case studies comes from the research design and its components, which Yin outlines as (a) the study’s questions, (b) the study’s propositions, (c) the unit of analysis, (d) the logic linking the data to propositions, and (e) the criteria for interpreting the findings [5]. Case studies should also provide multiple sources of data, a case study database, and a clear chain of evidence among the questions asked, the data collected, and the conclusions drawn [5].

**Figure 1:**
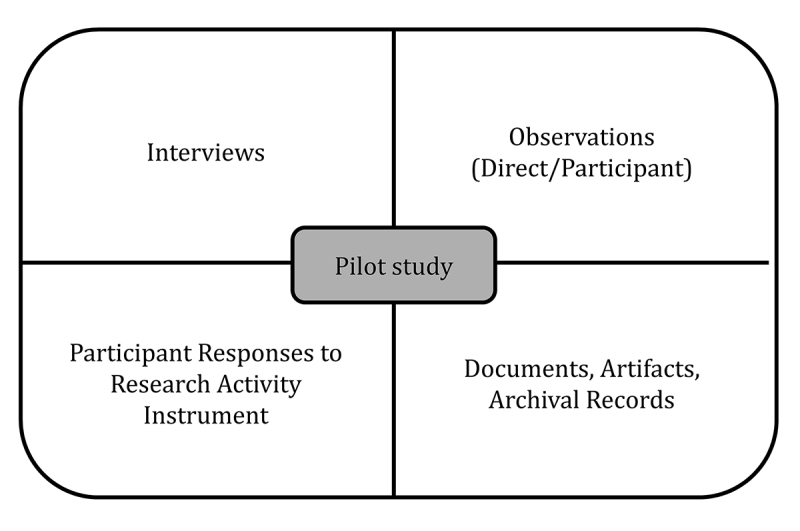
Key sources of data for a sample case study

Sources of evidence for case studies include interviews, documentation, archival records, direct observations, participant-observation, and physical artifacts. One of the most important sources for data in qualitative case study research is the interview [2, 3]. In addition to interviews, documents and archival records can be gathered to corroborate and enhance the findings of the study. To understand the phenomenon or the conditions that created it, direct observations can serve as another source of evidence and can be conducted throughout the study. These can include the use of formal and informal protocols as a participant inside the case or an external or passive observer outside of the case [5]. Lastly, physical artifacts can be observed and collected as a form of evidence. With these multiple potential sources of evidence, the study methodology includes gathering data, sense-making, and triangulating multiple streams of data. Figure 1 shows an example in which data used for the case started with a pilot study to provide additional context to guide more in-depth data collection and analysis with participants.

## VARIATIONS ON CASE STUDY METHODOLOGY

Case study methodology is evolving and regularly reinterpreted. Comparative or multiple case studies are used as a tool for synthesizing information across time and space to research the impact of policy and practice in various fields of social research [9]. Because case study research is in-depth and intensive, there have been efforts to simplify the method or select useful components of cases for focused analysis. Micro-case study is a term that is occasionally used to describe research on micro-level cases [10]. These are cases that occur in a brief time frame, occur in a confined setting, and are simple and straightforward in nature. A micro-level case describes a clear problem of interest. Reporting is very brief and about specific points. The lack of complexity in the case description makes obvious the “lesson” that is inherent in the case; although no definitive “solution” is necessarily forthcoming, making the case useful for discussion. A micro-case write-up can be distinguished from a case report by its focus on briefly reporting specific features of a case or cases to analyze or learn from those features.

## DATABASE INDEXING OF CASE REPORTS AND CASE STUDIES

Disciplines such as education, psychology, sociology, political science, and social work regularly publish rich case studies that are relevant to particular areas of health librarianship. Case reports and case studies have been defined as publication types or subject terms by several databases that are relevant to librarian authors: MEDLINE, PsycINFO, CINAHL, and ERIC. Library, Information Science & Technology Abstracts (LISTA) does not have a subject term or publication type related to cases, despite many being included in the database. Whereas “Case Reports” are the main term used by MEDLINE’s Medical Subject Headings (MeSH) and PsycINFO’s thesaurus, CINAHL and ERIC use “Case Studies.”

Case reports in MEDLINE and PsycINFO focus on clinical case documentation. In MeSH, “Case Reports” as a publication type is specific to “clinical presentations that may be followed by evaluative studies that eventually lead to a diagnosis” [11]. “Case Histories,” “Case Studies,” and “Case Study” are all entry terms mapping to “Case Reports”; however, guidance to indexers suggests that “Case Reports” should not be applied to institutional case reports and refers to the heading “Organizational Case Studies,” which is defined as “descriptions and evaluations of specific health care organizations” [12].

PsycINFO’s subject term “Case Report” is “used in records discussing issues involved in the process of conducting exploratory studies of single or multiple clinical cases.” The Methodology index offers clinical and non-clinical entries. “Clinical Case Study” is defined as “case reports that include disorder, diagnosis, and clinical treatment for individuals with mental or medical illnesses,” whereas “Non-clinical Case Study” is a “document consisting of non-clinical or organizational case examples of the concepts being researched or studied. The setting is always non-clinical and does not include treatment-related environments” [13].

Both CINAHL and ERIC acknowledge the depth of analysis in case study methodology. The CINAHL scope note for the thesaurus term “Case Studies” distinguishes between the document and the methodology, though both use the same term: “a review of a particular condition, disease, or administrative problem. Also, a research method that involves an in-depth analysis of an individual, group, institution, or other social unit. For material that contains a case study, search for document type: case study.” The ERIC scope note for the thesaurus term “Case Studies” is simple: “detailed analyses, usually focusing on a particular problem of an individual, group, or organization” [14].

## PUBLICATION OF CASE STUDY RESEARCH IN LIBRARIANSHIP

We call your attention to a few examples published as case studies in health sciences librarianship to consider how their characteristics fit with the preceding definitions of case reports or case study research. All present some characteristics of case study research, but their treatment of the research questions, richness of description, and analytic strategies vary in depth and, therefore, diverge at some level from the qualitative case study research approach. This divergence, particularly in richness of description and analysis, may have been constrained by the publication requirements.

As one example, a case study by Janke and Rush documented a time- and context-bound collaboration involving a librarian and a nursing faculty member [15]. Three objectives were stated: (1) describing their experience of working together on an interprofessional research team, (2) evaluating the value of the librarian role from librarian and faculty member perspectives, and (3) relating findings to existing literature. Elements that signal the qualitative nature of this case study are that the authors were the research participants and their use of the term “evaluation” is reflection on their experience. This reads like a case study that could have been enriched by including other types of data gathered from others engaging with this team to broaden the understanding of the collaboration.

As another example, the description of the academic context is one of the most salient components of the case study written by Clairoux et al., which had the objectives of (1) describing the library instruction offered and learning assessments used at a single health sciences library and (2) discussing the positive outcomes of instruction in that setting [16]. The authors focus on sharing what the institution has done more than explaining why this institution is an exemplar to explore a focused question or understand the phenomenon of library instruction. However, like a case study, the analysis brings together several streams of data including course attendance, online material page views, and some discussion of results from surveys. This paper reads somewhat in between an institutional case report and a case study.

The final example is a single author reporting on a personal experience of creating and executing the role of research informationist for a National Institutes of Health (NIH)–funded research team [17]. There is a thoughtful review of the informationist literature and detailed descriptions of the institutional context and the process of gaining access to and participating in the new role. However, the motivating question in the abstract does not seem to be fully addressed through analysis from either the reflective perspective of the author as the research participant or consideration of other streams of data from those involved in the informationist experience. The publication reads more like a case report about this informationist’s experience than a case study that explores the research informationist experience through the selection of this case.

All of these publications are well written and useful for their intended audiences, but in general, they are much shorter and much less rich in depth than case studies published in social sciences research. It may be that the authors have been constrained by word counts or page limits. For example, the submission category for Case Studies in the *Journal of the Medical Library Association (JMLA)* limited them to 3,000 words and defined them as “articles describing the process of developing, implementing, and evaluating a new service, program, or initiative, typically in a single institution or through a single collaborative effort” [18]. This definition’s focus on novelty and description sounds much more like the definition of case report than the in-depth, detailed investigation of a time- and space-bound problem that is often examined through case study research.

Problem-focused or question-driven case study research would benefit from the space provided for Original Investigations that employ any type of quantitative or qualitative method of analysis. One of the best examples in the *JMLA* of an in-depth multiple case study that was authored by a librarian who published the findings from her doctoral dissertation represented all the elements of a case study. In eight pages, she provided a theoretical basis for the research question, a pilot study, and a multiple case design, including integrated data from interviews and focus groups [19].

## CONCLUSION

We have distinguished between case reports and case studies primarily to assist librarians who are new to research and critical appraisal of case study methodology to recognize the features that authors use to describe and designate the methodological approaches of their publications. For researchers who are new to case research methodology and are interested in learning more, Hancock and Algozzine provide a guide [20].

We hope that *JMLA* readers appreciate the rigor of well-executed case study research. We believe that distinguishing between descriptive case reports and analytic case studies in the journal’s submission categories will allow the depth of case study methodology to increase. We also hope that authors feel encouraged to pursue submitting relevant case studies or case reports for future publication.
